# Preparation of Novel ICT-CMC-CD59sp Drug-Loaded Microspheres and Targeting Anti-Tumor Effect on Oral Squamous Cell Carcinoma

**DOI:** 10.3389/fbioe.2022.878456

**Published:** 2022-03-21

**Authors:** Xiang Gao, Wanchun Wang, Meihua Gao

**Affiliations:** ^1^ Department of Stomatology, School of Stomatology of Weifang Medical University, Weifang, China; ^2^ Qingdao Stomatological Hospital, Qingdao, China

**Keywords:** ICT-CMC-CD59sp microspheres, oral squamous cell carcinoma, CD59-specific ligand peptide, targeted antitumor effect, drug-loaded

## Abstract

The treatment of oral squamous cell carcinoma (OSCC) remains a great clinical challenge, and the malignant proliferation of OSCC cells can lead to the overexpression of CD59. In this study, a novel microsphere (ICT-CMC-CD59sp) composed of icariin (ICT), carboxymethyl chitosan (CMC), and cell differentiation antigen 59-specific ligand peptide (CD59sp) was successfully prepared by using the emulsion cross-linking method. Through the guidance of CD59sp, the microspheres can target OSCC cells and play a therapeutic role (*p* < 0.01). The MTT test and trypan blue staining showed that the microspheres could promote the apoptosis of oral squamous cell carcinoma and had a significant difference (*p* < 0.01). In this study, the regulatory effect of the microspheres on OSCC cells was investigated at the cellular level, and its therapeutic effect on OSCC was discussed, which provided a new perspective for the targeted therapy of OSCC.

## Graphical Abstract

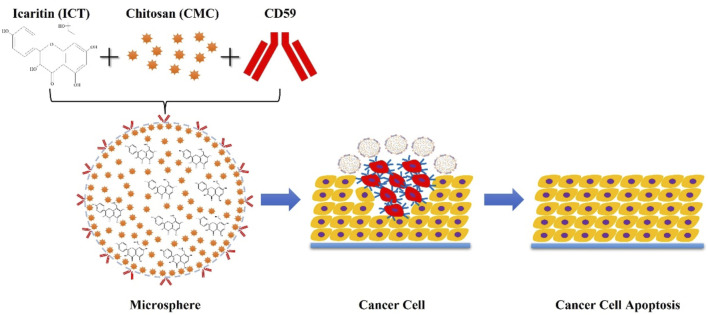

## Introduction

Oral cancer is the sixth most common malignant tumor in the world, with more than 350,000 cases every year, of which nearly 90% of oral mucosa and lip malignant tumors are diagnosed as oral squamous cell carcinoma (OSCC) (Bray et al., 2018; Panarese et al., 2019). Common sites of OSCC are the tongue and floor of the mouth (Duray et al., 2012; Kouketsu et al., 2016). OSCC results in ulcerative lesions in the lesion area, with central necrosis and surrounding bulges (Pires et al., 2013). It mainly affects men aged 50–60 years, but the incidence rate of young patients has increased in recent decades (Tsimplaki et al., 2014; Kouketsu et al., 2016). The occurrence of OSCC may be caused by pre-existing oral lesions, and smoking and drinking are also important risk factors for OSCC (Dotto and Rustgi, 2016; Cramer et al., 2019). At present, the World Health Organization believes that oral leukoplakia, erythroplakia, oral submucosal fibrosis, reverse smoking palatal lesions, and oral lichen planus will increase the risk of cancer progression (Vitorio et al., 2020).

The treatment of OSCC has always been a difficult problem in clinics. At present, radiotherapy, chemotherapy, surgery, and anticancer drugs are still the main methods for the treatment of oral cancer, but these methods still have serious side effects (Sivanantham et al., 2016). Therefore, it is urgent to adopt new methods to change the treatment of oral cancer. In recent years, the rapid development of biomedical materials has shown a great progress in cancer treatment. At present, due to the enhancement of targeting and specificity of biomaterials, many drug-loaded biomaterials have shown great tumor cell killing ability. Among them, tumor targeted therapy has high specificity, which can accurately deliver drugs to tumor cells, reduce the toxic and side effects of drugs, prolong the half-life of drugs *in vivo* and enhance the curative effect (Masood, 2016). Therefore, it is imperative to explore new targeted therapies.

In recent years, many studies have been carried out on the anticancer effect of icaritin (ICT) (Zhang et al., 2018; Zhou et al., 2018; Wu et al., 2020; Tao et al., 2021), the main active component of *Epimedium*. For example, studies have shown that ICT can inhibit cell viability, migration, and invasion and induce apoptosis by downregulating miR-625–3p and inactive PI3K/AKT and MEK/ERK signaling pathways in thyroid cancer cells (Fang et al., 2019). Another study showed that ICT can delay tumor progression, reduce the percentage of myelogenous suppressor cells (MDSCs) and have immunosuppressive function (Gabrilovich and Nagaraj, 2009). Previous studies by our research group also found that ICT could induce tumor cell apoptosis and inhibit tumor cell invasion (Wang et al., 2021). However, if ICT is used as a single component in cancer treatment, its bioavailability will be very low (2%), so it is necessary to organically combine ICT with a drug carrier capable of drug delivery for cancer treatment. Carboxymethyl chitosan is a good marine drug carrier, which can wrap a variety of antitumor drugs, prolong the drug action time, reduce side effects, and improve the drug efficacy. CD59 is a membrane complement regulatory protein, which is highly expressed in many reproductive system tumors and is related to tumor immune deficiency (Sivasankar et al., 2009). Therefore, in this study, triad-targeted drug delivery microspheres (ICT-CMC-CD59sp) were prepared with ICT as the antitumor model drug, CMC as the carrier, and CD59sp as the targeting molecule to explore its antitumor effect, which opened up a new way for targeted treatment of OSCC, which has important clinical value.

## Materials and Methods

### Materials

Icaritin (ICT) was purchased from Tauto Biotech (Shanghai, China); carboxymethyl chitosan (CMC, CAS 83512–85-0), MTT cell proliferation and cytotoxicity assay kits, and trypan blue staining cell viability assay kits were purchased from Solarbio (Beijing, China); CD59 peptide ligands were purchased from Chinese Peptide (Hangzhou, China); Human TNF-α ELISA kits were purchased from JiuBang Biotech (Quanzhou, China); N-(3-dimethylaminopropyl)-N′-ethylcarbodiimide hydrochloride (EDC) was purchased from Sigma-Aldrich (Shanghai, China); and oral squamous cell carcinoma (OSCC-9) was purchased from Procell (Wuhan, China).

### Detection of CD59 on Cell Surface of Oral Squamous Cell Carcinoma

Paraffin sections were placed in fresh xylene, soaked for 10 min, and repeated once. After removing the excess liquid, the sections were placed in absolute ethanol, soaked it for 3 min, and repeated it once. After removing the excess liquid, the sections were soaked in 95% ethanol for 3 min and repeated once. After removing the excess liquid, the sections were soaked it in 75% ethanol for 3 min and repeated it once. The sections were washed with distilled water for 1 min (wash away the alcohol) and were placed in PBS buffer for 1–3 min. Then microwave antigen repair was carried out for 2 min and cooled to room temperature. After that, an appropriate amount of endogenous peroxidase blocker was added to the sections, washed with PBS buffer for 2 min, and repeated 3 times. Primary antibody was added, incubated overnight at 4°C, washed in PBS buffer for 2 min, and repeated twice; 100 μl reaction enhancer was then added, incubated at room temperature for 20 min, washed with PBS for 2 min and repeated twice; goat anti-mouse/rabbit IgG polymer with enhanced enzyme labeling was added; followed by DAB chromogenic solution, incubated at room temperature for 5 min, and rinsed with tap water. Hematoxylin counterstaining was performed, incubated with staining solution for 12 s, washed with tap water for 5 min, followed by differentiation washing and anti-blue, gradient dehydration, transparent, drip neutral gum seal, and finally the film was read.

### Preparation of Microspheres

In total, 120 mg of carboxymethyl chitosan powder was added to 7.5 ml of aqueous solution and stirred evenly to form the aqueous phase. Then 37.5 ml of liquid paraffin and 3 ml of EL35 was added to a 50-ml beaker and stirred evenly to form the oil phase. After mixing the aqueous and oil phases, the mixture was stirred at room temperature at 1,000 r/min, allowing it to emulsify for 70 min. After that, 0.25 ml of 50% glutaraldehyde was added to the emulsion, which was left for 150 min for cross-linking and curing, static, and abandoning the upper emulsion. The mixture was then washed with dioxane, acetone, and anhydrous ethanol, followed by drying by filtration. A brown powder was obtained as CMC microspheres, was stored at 4°C for standby.

In total, 6 mg ICT was taken and dissolved in 3 ml anhydrous ethanol, and then ultrasound was carried out for 30 min to fully dissolve it. Centrifugation was performed at 8,000 r/min. After that, the solution was discarded, the supernatant was taken to dissolve it, making 2 mg/ml of ICT ethanol solution, and 3 ml of the solution was taken and added to 7 ml of chitosan aqueous solution. Then 37.5 ml liquid paraffin and 3 ml EL35 were added to a 50-ml beaker and stirred evenly at a speed of 1,000 r/min to form an oil phase. The prepared ICT solution was added to the oil phase, stirred at room temperature, left to emulsify for 70 min, and then 0.25 ml 50% glutaraldehyde was added to the emulsion and left 150 min to cross-link. After that, the upper emulsion was discarded and cleaned with dioxane, acetone, and anhydrous ethanol. After suction filtration and drying, the final brown powder was an ICT-CMC microsphere, which was stored at 4°C for standby.

In total, 5 mg of ICT-CMC microspheres were taken, resuspended in PBS, and thoroughly mixed. To the mixture, 2 mg of EDC was added, the pH of which was adjusted to 5.6, stirred at room temperature for 1 h, and centrifuged at 8,500 rpm for 10 min, and the unreacted EDC was then removed. Then the mixture was resuspended in 1 ml PBS, 1 mg/ml FITC-labeled CD59sp was added to it, stirred overnight, centrifuged to remove unbound CD59sp, resuspended in 1 ml PBS, and lyophilized into powder, namely, ICT-CMC-CD59sp microsphere, which was stored at 4°C for standby.

### Characterization

The microstructure of the microsphere was imaged using a scanning electron microscope (SEM, JSM-5600, Japan). An appropriate number of microspheres were taken and directly stuck to the conductive adhesive surface. After spraying gold, it was observed and photos were taken under a scanning electron microscope. Fourier Transform Infrared Spectroscopy (FTIR) characterization and detection were carried out as follows: an appropriate number of ICT-CMC-CD59sp microspheres were taken, ground into powder, and then were detected with an infrared spectrometer with a scanning range of 500–4,000 cm^−1^.

### *In Vitro* Anticancer Assay

#### Cell Experiment

Cell experiments were divided into three groups: the CMC microsphere group, the ICT-CMC microsphere group, and the ICT-CMC-CD59sp microsphere group. The microsphere concentrations of each group in this part were 2 mg/ml, 1 mg/ml, and 0.5 mg/ml, respectively.

#### MTT Assay for Cytotoxicity

The effect of microspheres on the proliferation of OSCC cells is as follows:

1) Logarithmic growth stage cells were collected, and cell suspension concentrations were adjusted to 4 × l0^4^ ml, 100 μl of which were added to each well.
2) The cells were cultured at 37°C and 5% CO_2_ environment for 24 h to adhere to the wall.
3) Different concentrations of 100 μl of different types of microspheres were added and cultured for 24 h.
4) The supernatant was removed, which was added to 90 μl fresh culture solution, and 10 μl MTT solution, and then cultured for 4 h.
5) The supernatant was removed. Then 110 μl Formazan solution was added to each well and shaken on a shaking table at low speed for 10 min to fully dissolve the crystals. The absorption value of each was measured at 490 nm by using an enzyme immunoassay apparatus.

#### Trypan Blue Staining

The process of trypan blue staining is as follows: 4 g of trypan blue was taken and a small amount of distilled water was added for grinding. Double distilled water was added to 100 ml to prepare 4% trypan blue liquor, filtered, and stored at 4°C, which was diluted to 0.4% with PBS. Adherent OSCC cells were digested with trypsin to prepare a single cell suspension and diluted appropriately. The cell suspension and 0.4% trypan blue solution were mixed evenly at 9:1 (final concentration 0.04%). For the stained cell materials, a drop of cell suspension was taken on a glass slide, then the glass slide was covered, and observed under a high-power microscope. The number of living cells and dead cells were counted within 3 min. The dead cells were light blue, swollen, and dull. The living cells do not stain and maintain their normal shape and luster. Statistical staining cells and cell mortality were calculated (Cell mortality = number of stained cells/total number of observed cells×100%).

#### Enzyme-Linked Immunosorbent Assay Test: Detect TNF-α in Cell Supernatant Level

The cell supernatant of different groups of microspheres were taken after 24 h and centrifuged at 1,000×*g* for 20 min. The required strips were taken out from the aluminum foil bag after 20 mins of room temperature balance, and the remaining strips were sealed with natural sealing bags and put back at 4°C. The standard hole and sample hole were set, then 50 μl of each of the 80, 40, 20, 10, 5 and 2.5 pg/ml standard samples were added to the standard hole. Then 50 μl samples were added to the hole to be tested, and the blank was not added. In addition to the blank, 100 μl of horseradish peroxidase (HRP)-labeled detection antibody was added to each standard hole. The reaction hole was sealed and incubated in the incubator for 60 min. After washing the plate for 5 times, 50 μL substrates A and B were added to each well and incubated in the dark at 37°C for 15 mins. Then 50 μl termination solution was added to each well. The OD value of each hole was measured at a wavelength of 450 nm within 15 min.

### Statistical Analysis

We used OriginPro 8 and GraphPad Prism8 to process the image analysis data, and the values were expressed as mean ± standard deviation. In the analysis of variance between groups, *p* < 0.05 was considered statistically significant.

## Results

### CD59 Expression in Oral Squamous Carcinoma Tissue Cells

**FIGURE 1:**
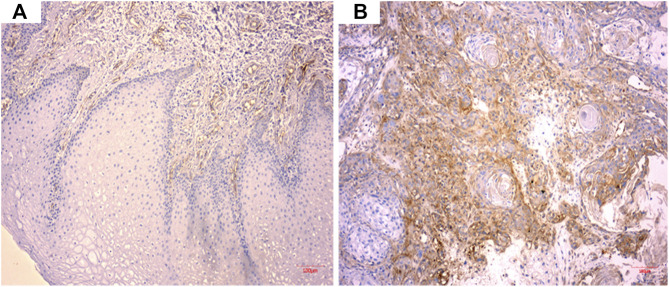
CD59 expression in oral squamous cancer cells. **(A)** Low expression of CD59 in normal oral keratinocytes. **(B)** High expression of CD59 in oral squamous carcinoma cells.

As shown in Figure 1, CD59 was overexpressed in oral squamous carcinoma cells compared with normal keratinocytes.

### Comparison of Synergistic Antitumor Effects of ICT/CMC/CD59sp

**FIGURE 2:**
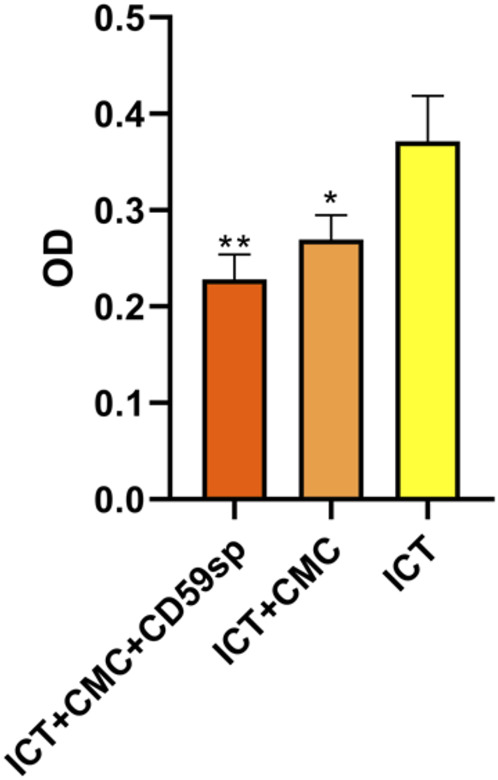
Comparison of the synergistic antitumor effects of ICT/CMC/CD59sp (*p < 0.05, **p < 0.01).

As shown in Table 1 and Figure 2, the ICT-CMC-CD59sp group (*p* < 0.01) and ICT-CMC group (*p* < 0.01) have significant differences compared with the ICT group, indicating that ICT-CMC-CD59sp and ICT-CMC have better synergistic effects and antitumor effects than ICT.

**TABLE 1:** Comparison of synergistic antitumor effects of ICT/CMC/CD59sp.

Group	OD (‾x ± *s*)	*p*
ICT	0.371 ± 0.047	-
ICT + CMC	0.269 ± 0.025*	<0.05
ICT + CMC + CD59sp	0.228 ± 0.026**	<0.01

### Scanning Electron Microscope Observation Results

**FIGURE 3:**
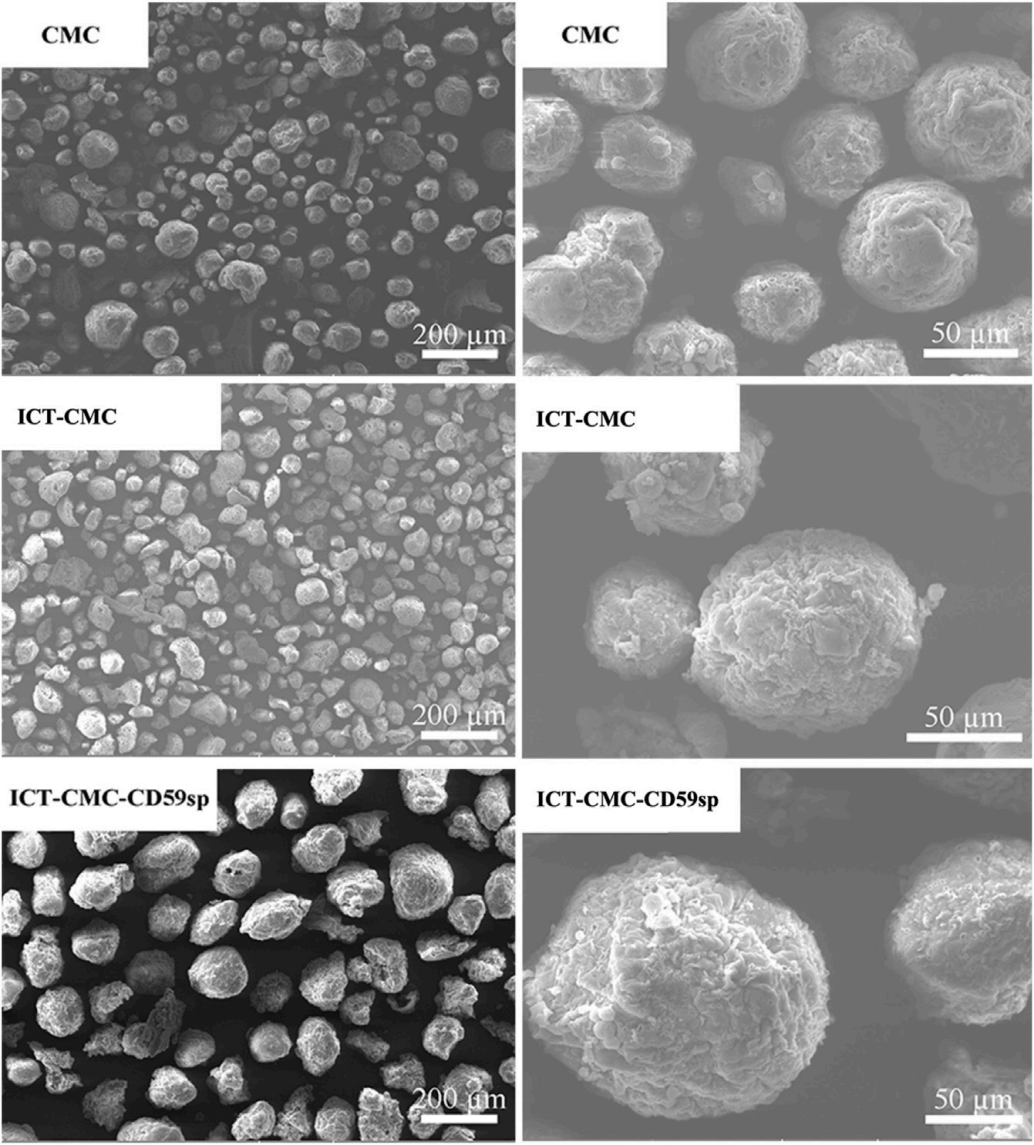
Images of CMC, ICT-CMC, and ICT-CMC-CD59sp microspheres.

Figure 3 shows the SEM images of CMC microspheres, ICT-CMC microspheres, and ICT-CMC-CD59sp microspheres, respectively. It can be seen from the figure that the microspheres show regular spheres, evenly dispersed and with clear pores.

### FTIR Characterization

**FIGURE 4:**
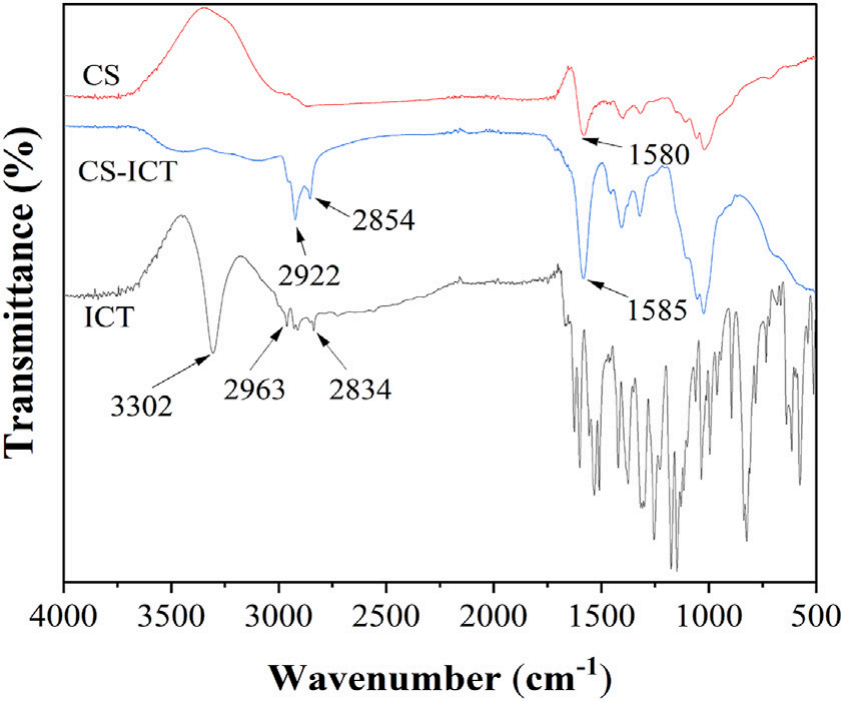
Infrared spectrum images of ICT, CMC, and ICT-CMC microspheres.

Figure 4 shows the infrared analysis of ICT, CMC, and ICT-CMC microspheres; 3,302 cm^−1^ is the stretching vibration peak of O–H in ICT, 2,963 cm^−1^ and 2,834 cm^−1^ are the stretching vibration peaks of C–H in CH2 and CH3, and the corresponding is the characteristic peak of ICT. The corresponding peaks of 1,580 cm^−1^ and 1,585 cm^−1^ were characteristic peaks of amide in chitosan. After drug loading, characteristic peaks of ICT and CMC were found in ICT-CMC microspheres at the same time, indicating that ICT was successfully loaded into CMC microspheres.

### Drug-Loaded Microspheres Targeting Combining With Cell Surface

**FIGURE 5:**
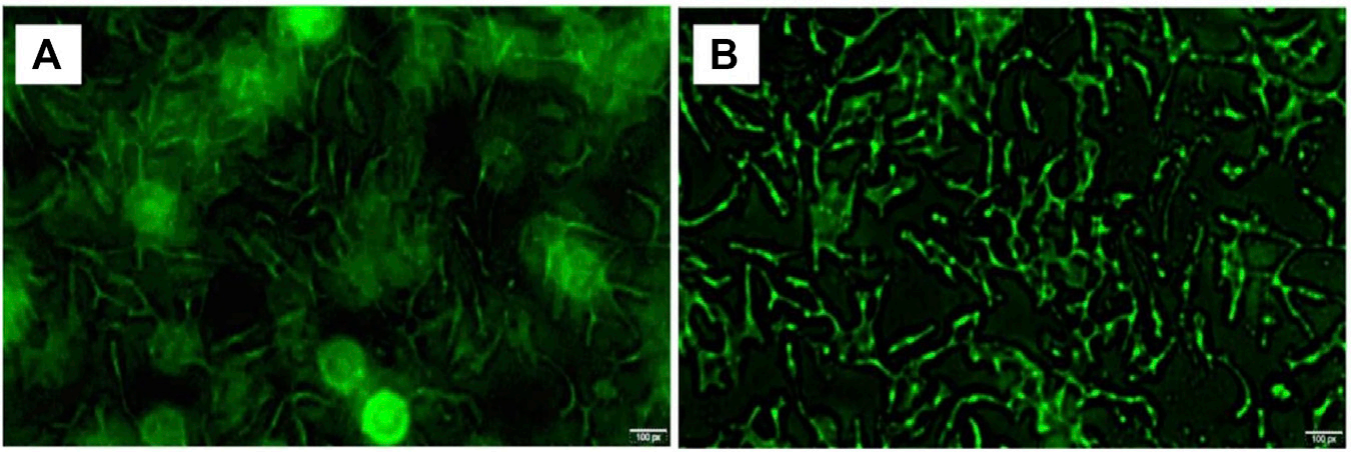
Image of high expression of CD59 on oral squamous cell carcinoma cells **(A)** and ICT-CMC-CD59sp targeting microspheres’ killing effect **(B)**.

OSCC cell surface fluorescence intensity (Figures 5A, B) shows that OSCC cells wrinkled into bar cord by using a fluorescence microscope 24 h after ICT-CMC-CD59sp targeting microspheres. Thus, it can be seen that CD59sp can specifically bind OSCC-9 cell CD59 molecules and deliver ICT to OSCC-9 cells with high penetration of CMC, jointly killing oral squamous cancer cells, thus exerting a good synergistic antitumor effect.

### Inhibition Effect of Microspheres on Keratinocytes and OSCC-9 Cells at Different Concentrations

**FIGURE 6:**
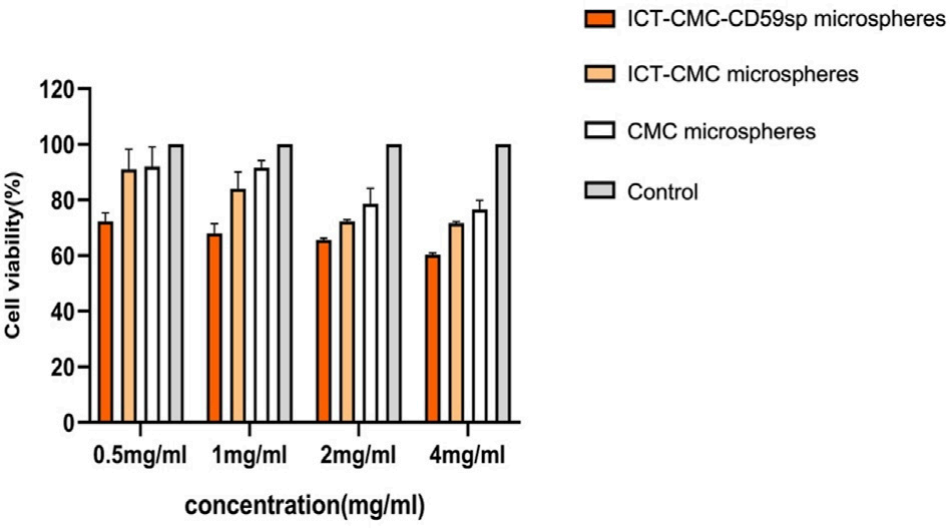
Effect of ICT-CMC-CD59sp on keratinocytes.

**FIGURE 7:**
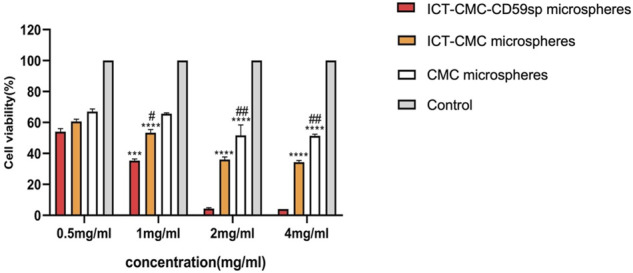
Killing effect of microspheres on OSCC-9 cells (ICT-CMC-CD59sp group compared with other groups, ^***^ *p* < 0.001; CMC group compared with ICT-CM group, ^#^ *p* < 0.05, ^##^ *p* < 0.01).

As shown in Figures 6, 7, microspheres containing different components and concentrations of 0.5 mg/ml, 1 mg/ml, 2 mg/ml, and 4 mg/ml were used to detect the effects on keratinocytes and OSCC-9 cells. As shown in Figure 6, different microspheres had little effect on the keratinocytes. However, ICT-CMC-CD59sp microspheres had a great inhibitory effect on the growth of tumor cells. Among them, 4 mg/ml and 2 mg/ml ICT-CMC-CD59sp microsphere groups had the best effects. Since there is no significant difference between the two groups, 2 mg/ml is selected for subsequent experiments. There was a significant difference between the ICT-CMC-CD59sp microsphere group and the ICT-CMC microsphere group and the CMC microsphere group (*p* < 0.01). The aforementioned results showed that the ICT-CMC-CD59sp group had a good antitumor effect in a concentration-dependent manner.

### Oral Squamous Cell Carcinoma Trypan Blue Staining Detects Cell Mortality of OSCC-9 Cells

According to the analysis of Table 2, the ICT-CMC-CD59sp microsphere group had the best effect compared with the ICT-CMC microsphere group and the CMC empty microsphere group (*p* < 0.01).

**TABLE 2:** Comparison of trypan blue staining results in living cells.

Group	Cell mortality (%) (‾x ± *s*)	*p*
CMC	10 ± 7.65^***^	<0.01
ICT-CMC	54.18 ± 4.1^**^	<0.01
ICT-CMC-CD59sp	71.5 ± 6.64	-

### Effects of Different Microspheres on Tumor Necrosis Factor in SCC-9 Cells

**FIGURE 8:**
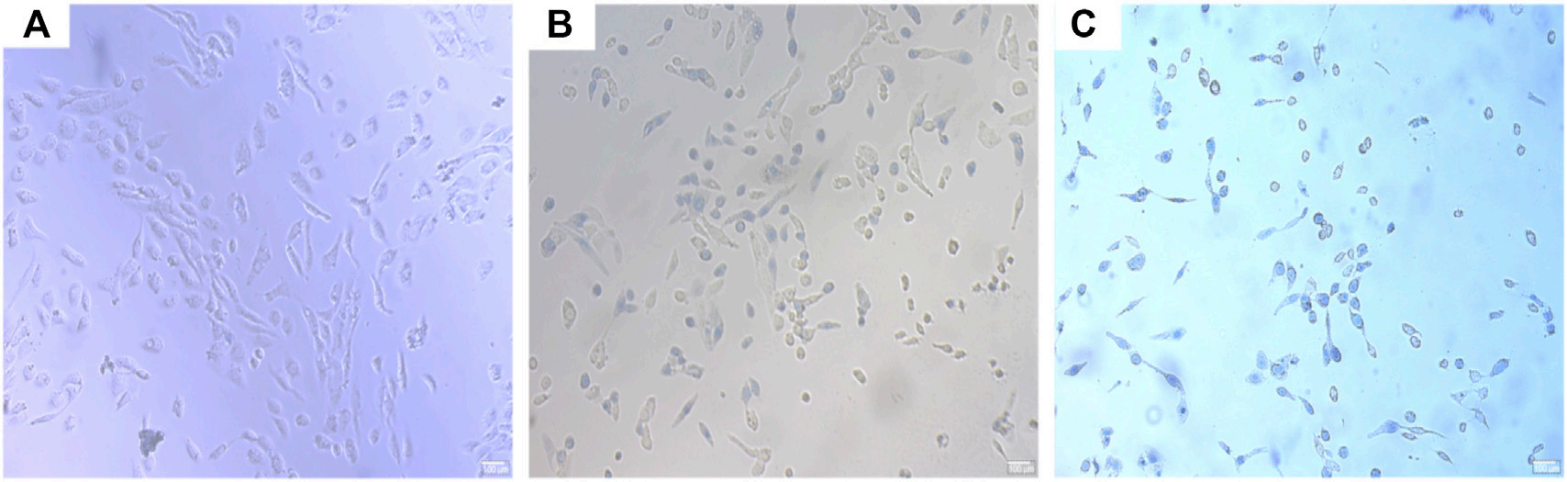
OSCC trypan blue staining. **(A)** CMC microsphere group, **(B)** ICT-CMC microsphere group, and **(C)** ICT-CMC-CD59sp microsphere group.

**FIGURE 9:**
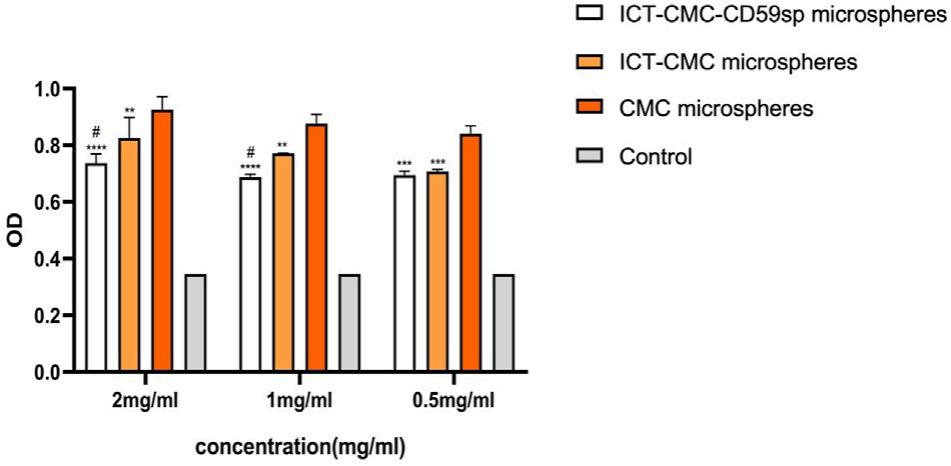
Effect of ICT-CMC-CD59sp targeting of microspheres on tumor necrosis factor-α (different concentrations of each group compared with ICT-CMC-CD59sp microsphere group, ^**^ *p* < 0.01, ^***^ *p* < 0.001; CMC microsphere group compared with ICT-CMC microsphere group, ^#^ *p* < 0.05).

The results of ELISA showed that each microsphere group could promote the secretion of tumor necrosis factor *α* (Figures 8, 9). Compared with the ICT-CMC microsphere group and the CMC microsphere group, the ICT-CMC-CD59sp microsphere group with 2 mg/ml had the best effect (*p* < 0.01).

## Discussion

In recent years, research into targeting drug delivery systems has become a focus in the oncology field. Targeted drugs can deliver drugs to the targeted sites, reduce drug toxicity, prolong circulation half-lives, increase bioavailability, and enhance tumor disposition (Arranja et al., 2017).

Carboxymethyl chitosan is a water-soluble polysaccharide with higher solubility, moisture retention, and adsorption (Qing Chen et al., 2006; Shariatinia, 2018; Xu et al., 2018). It has stronger antibacterial properties, biocompatibility, and safety for the human body (Liu et al., 2005; Liuyun et al., 2009; Jayakumar et al., 2010). It has been gradually used as a controlled release carrier of drugs in the biomedical field in recent years (Zhou et al., 2004; Ray-Neng Chen et al., 2006; Wang et al., 2010). Yang et al. (2017) prepared carboxymethyl chitosan nanoparticles (CMCNPs) coated with phycocyanin (C-PC) in an ion cross-linking method, and their cell experiments confirmed the obvious inhibitory effect of their microspheres on Hela cells. Hefnawy et al. (2020) target ASGP receptors highly expressed on the tumor cell surface and deliver DOX complexed with carboxymethyl chitosan-g-poly (acrylate) to achieve the targeted killing of HCC cells.

As the active ingredient of *Epimedium*, ICT has a good curative effect. It can not only promote blood circulation and enhance immunity and cardiac function but also induce apoptosis, downregulate tumor angiogenesis, and inhibit human cancer cell invasion of prostate cancer cells and endometrial cancer cells. In addition, ICT can promote tumor necrosis factor-*α* so as to promote the apoptosis of cancer cells (Li et al., 2013; Zhu et al., 2015; Wu et al., 2020). However, its stability is poor and is easily degraded, and its bioavailability is low.

The previous study found that CD59 molecules are overexpressed in various reproductive system tumors (cervical cancer, prostate cancer, breast cancer, etc.) (Zhang et al., 2018; Wang and Liang, 2020), but the correlation between CD59 and oral squamous cancer has not been reported. In this study, we targeted squamous carcinoma of the oral cavity using CMC as the vector to prepare an ICT-containing microsphere and connected CD59sp to the microsphere by EDC activation to obtain the targeted drug-carrying microsphere, CD59 ligand peptide, and CD59 protein on the tumor cell surface to achieve drug targeting.

The results showed that the microspheres had good dispersibility. By Scanning electron microscopy, the particle size of the microspheres was about 100 μm, and the microspheres were spherical by electron microscopy. The results of the MTT assay and trypan blue dyeing showed that microspheres inhibited the proliferation of OSCC cells, and there were significant differences between groups at different concentrations (*p* < 0.01), among which the ICT-CMC-CD59sp group had the best inhibition effect.

Mechanism of the anti-tumor effect of ICT-CMC-CD59sp microspheres: The overexpression of CD59 suppresses the formation of the membrane attack complex (MAC), by which tumor cells can avoid recognition by the complement pathway (Huang et al., 2002). Our microspheres can bind specifically to the CD59 receptor of tumor cell CD59 led by CD59sp, guide drugs to reach the cell surface, activate complement to form MAC, and dissolve OSCC tumor cells, and then, through receptor-mediated endocytosis, the drug were taken to the cells’ interior. The ICT-CMC-CD59sp microspheres constructed in this experiment can bind CD59sp specifically to the tumor cell CD59 to guide the drug to the surface of oral squamous cancer cells, intake Epanin inside the cells, activate the complement to form MAC and lyse OSCC tumor cells. TNF-α can also bind to TNF-receptors and then activate the caspase protease family, promote caspase-3 activation, and induce death of oral squamous cancer cells.

## Conclusion

In conclusion, this study optimized the preparation conditions of ICT-CMC-CD59sp and successfully made the ICT-CMC-CD59sp triad of targeted drug delivery microspheres with high efficiency and sustained-release performance. It was confirmed that the microspheres could inhibit the proliferation of OSCC cells, providing a new perspective and approach for the application of ICT-CMC-CD59sp in the field of oral tumors, which has important theoretical significance and clinical application value.

## Data Availability

The raw data supporting the conclusion of this article will be made available by the authors, without undue reservation.
